# Boeravinone B alleviates gut dysbiosis during myocardial infarction‐induced cardiotoxicity in rats

**DOI:** 10.1111/jcmm.16620

**Published:** 2021-05-24

**Authors:** Yu Chen, Lei Peng, Shaoqing Shi, Gang Guo, Heling Wen

**Affiliations:** ^1^ Department of Cardiology Sichuan Academy of Medical Science &Sichuan Provincial People's Hospital Chengdu China; ^2^ Department of Nephrology Sichuan Academy of Medical Science & Sichuan Provincial People's Hospital Chengdu China; ^3^ Department of Pulmonary and Critical Care Medicine The First Affiliated Hospital of Kunming Medical University Kunming China; ^4^ Department of Talent Highland First Affiliated Hospital of Xi’an Jiao Tong University Xian China

**Keywords:** apoptosis, Boeravinone B, gut microbiota, isoproterenol, myocardial infarction

## Abstract

Myocardial infarction (MI) is the most common heart disease, and also, it is one of the leading causes of death from cardiovascular disease. It is well known that MI causes additional injury during blood flow restoration in ischaemic myocardium. Boeravinone B (BB) is a well‐known antioxidant and anti‐inflammatory drug. We investigated the cardioprotective effect of BB drug against isoproterenol (ISO)‐induced MI in rats in this experimental study, along with we analysed its underlying mechanism. Adult Sprague Dawley (SD) rats were treated subcutaneously with ISO (45 mg/kg), then divided into groups and then given BB drug was administered orally. The cardioprotective effect of BB on ISO‐induced MI rats was analysed by estimating the heart injury markers, antioxidant pro‐inflammatory cytokines and inflammatory parameters. We also detected quantified expression of inflammation and apoptosis‐related marker protein family. We estimated the effect of BB drug on GUT microbiota in ISO‐induced MI rats and scrutinized the histopathological variations in heart tissues. BB treatment significantly (*P* < .001) diminished the level of heart markers such as lactate dehydrogenase (LDH), troponin (TnT), creatine kinase (CK) and creatine kinase isoenzymes MB (CK‐MB). BB treatment also altered the antioxidant parameters and reduced the pro‐inflammatory cytokines in the serum and tissues. Additionally, the histopathological aspects demonstrated that the pathological changes observed in the heart tissue of the ISO group rats were suppressed by the BB treatment to varying degrees. Furthermore, the expressions of caspase‐3, p53, caspase‐9, Bax, interleukin‐6 (IL‐6), cytochrome C, neutrophil gelatinase‐associated lipocalin (NGAL), tumour necrosis factor‐α (TNF‐α), nuclear factor kappa B (NF‐κB) and interleukin‐1β (IL‐1β) in the heart tissue were down‐regulated whereas the Bcl‐2 expression seemed to be enhanced. BB treatment not only alleviated ISO‐induced gut dysbiosis by its enhanced specified *Firmicutesto‐Bacteroidetes* (F/B) ratio but also maintained the relative abundance of major bacteria such as Clostridium IV, Butyricicoccus, Clostridium XIVs, Akkermansia and Roseburia. Collectively, our findings showed that the BB drug acted against myocardial infraction and prevented the damage by reducing the oxidative stress and controlling the inflammatory pathways, and gut microbiota.

## 1 INTRODUCTION

Acute myocardial infarction is considered to be significant of ischaemic heart disease and it widely causes mortality and morbidity worldwide.^1^, ^2^ Myocardial infarction (MI) is a diseased condition which is generated as a result of the induction of necrosis that occurs as a result of imbalance between the myocardial demand and coronary blood supply. MI is an adverse health issue and it is one of the leading causes of higher mortality and morbidity rates in the Western world, including China.^2^, ^3^ MI‐related morbidity and mortality have reached epidemic proportions, with 16.7 million deaths per year worldwide.^2^ The clinical symptoms of myocardial dysfunction and necrosis include electrocardiographic changes, changes in blood pressure, left ventricular dysfunction and changes in heart beat rate. These symptoms arise as a result of the boosting of cardiac specific proteins level in the circulation.^4^, ^5^ The level of cardiac troponins frequently correlated with myocardial inflammation and infarction‐related proteins that increased the risk of heart attack. Moreover, MI also induces cardiac hypertrophy and myocardial fibrosis. The specific mechanism involved in MI has been associated with inflammation, oxidative stress and apoptosis.^6^, ^7^

Isoproterenol (ISO)‐induced myocardial infarction is considered to be a significant technique for inducing stress and inflammation in the rat's heart and also it aids in the estimation of cardioprotective effect of the tested compound. ISO (1‐[3,4‐dihydroxyphenyl]‐2‐isopropylamino ethanol hydrochloride) is a synthetic β‐adrenergic catecholamine which is used in the infra maximal doses to regulate the heart function.^3^, ^8^ Supramaximal dose of ISO could induce stress in the myocardium by reducing the amount of stored energy molecules in cardiomyocytes, which ultimately induces the infarct such as necrosis and irreversible cellular damage.^9^, ^10^ Various synthetic drugs have been used for treating this condition of heart attack, but most of the treatments show more severe side effects which may be fatal for patients. Nowadays scientists are focussing mainly towards plant‐based drugs to scrutinize the cardioprotective effect against myocardial infarction. Any natural compound with good antioxidant activity is thought to protect cardiac tissues from MI. Studies suggest that ISO‐induced myocardial dysfunction in the heart of the rodent is similar to human myocardial ischaemia.^4^, ^11^ As a result, ISO is widely used to induce MI in rats to investigate the effects of different processed drugs on MI.

Oxidative stress is involved in damaging the myocardial structure during cardiac hypertrophy. Additionally, evidence suggests that oxidative stress is an indicative of reduced levels of endogenous antioxidants in myocardium.^5^, ^8^ During oxidative stress, the activity of endogenous antioxidants such as catalase (CAT), glutathione peroxidase (GPx) and superoxide dismutase (SOD) was seen to be greatly decreased leading to the induction of lipid peroxidation. Increased levels of lipid peroxides activated malondialdehyde (MDA) which boosted the production of free radicals thereby suppressed the endogenous antioxidant status. It is well recognized that ISO induces the generation of reactive oxygen species (ROS) or free radical, which further causes oxidative stress, as demonstrated by considerably boosted the tissue MDA (lipid peroxidation marker) and suppressed the level of endogenous antioxidant enzymes such CAT, GPx and SOD, which have significant role against myocardial infarction.^2^, ^3^, ^12^ Previous studies suggested that the myocardial infarct size can be limited by enhancing the endogenous antioxidants level that reduces the generation of free radicals. During oxidative stress condition, the production of ROS and the secretion of pro‐apoptotic factors are initiated by mitochondria into the cytosol which activates the apoptotic cell death and signs of endoplasmic reticulum stress.^1^, ^13^, ^14^

The inflammatory response is essential in the progression of MI. Previous investigations suggest that pro‐inflammatory cytokines damage the myocardial tissue. Moreover, MI effects are elevated as a result of an increase in the level of pro‐inflammatory cytokines which also lead the tissue infiltration through inflammatory cells.^14^, ^15^ During MI, neutrophils invade the infarcted region, where they induce and increase myocardial cell injury by secreting proteolytic enzymes, a variety of chemokines and inflammatory cytokines, along with the production of reactive oxygen species (ROS).^6^, ^14^ Chemokines, reactive oxygen species and cytokines all contribute to the loss of organ function. A rise in pro‐inflammatory cytokines speeds up MI, which leads to serious congestive heart failure. IL‐1β, TNF‐α and IL‐6 are pro‐inflammatory cytokines that increase the inflammatory response during MI disease. TNF‐α causes neutrophil migration into the ischaemic region of infarcted myocardial tissue. Other cytokines involved in the stress‐induced inflammatory responses in myocardial tissue include IL‐6 (pleiotropic cytokine).^7^ MI‐attributed adverse reaction is categorized by the activation of various cellular signalling molecules, such as nuclear factor kappa light chain boost‐activated B cells (NF‐κB). It is proved that apoptosis and inflammatory reactions are regulated by the NF‐kB. In the MI diseased condition, NF‐κB phosphorylation initiates the intracellular signalling reaction and ultimately causes the generation of pro‐inflammatory cytokines and inflammation‐related proteins that involve numerous pathophysiological alterations.^7^, ^14^

The gut microbiota is composed of five phyla such as Proteobacteria, Firmicutes, *Bacteroidetes, Cerromicrobiota* and *Actinobacteria*. Among the five phyla of microbiota, anaerobic gram‐positive *Firmicutes,* especially Clostridium and Lactobacillus, and anaerobic gram‐negative Bacteroidetes, especially the *Prevotella* and Bacteroides, occupy more than 90% of gut microbial community, although the ratio of Firmicutes to Bacteroidetes varies from person to person. The difference in the microbial diversity is due to various host genomes and other factors such as diet, lifestyle variation, hygiene, excessive use of antibiotics, cancer and usage of drugs. Other phyla, such as fungi and archaea, constitute less than 1% of the gut flora.^6^, ^16^ These microorganisms in the gut convert carbohydrates and proteins into the various short chain fatty acids (SCFAs). The generated metabolites such as propionates (formed via Bacteroidetes), butyrates (formed via Firmicutes) and acetates degrade indigestible polysaccharides, which are then consumed and digested in the gut's distal region. Therefore, gut flora use the host's dietary derived molecules and considerably subsidize to gastrointestinal tract (GIT) physiological homeostasis, but any alteration in the gut dysbiosis (micro‐ecosystem) may pave a way for the pathophysiological function of host cardiovascular system.^17^ The gut microbiota controls the host's toxicity response, according to evidence pertaining to the gut. In cardiovascular patients, ISO causes gut dysbiosis, although the relationship between these microbiota changes and the magnitude of these side effects is still unknown.^16^ Dysbiosis of the gastrointestinal microbiota elevates the levels of gut‐derived toxins in the bloodstream, boosts the oxidative stress and inflammation and also affects intestinal absorption and utilization of micronutrient.^16^, ^18^

Boeravinone B is a rotenoid isolated from the *Boerhaavia diffusa*.^19^ The herb has been long in use by our ancestors and proved to be an effective formula for gastric ailments such as dyspepsia, abdominal pain, gastritis and other gut‐related disorders. Boeravinone B and C exhibited the P‐gp inhibitory effect.^19^ Another member of the rotenoid family such as Boeravinone G shows the antioxidant and genoprotective effect.^20^ BB showed the various pharmacological effects for the treatment of acute myoskeletal dysfunctions, spondylosis, rheumatoid arthritis, osteoarthritis, systemic lupus erythematosus, psoriasis and atherosclerosis in rodents.^20^ BB showed the anti‐inflammatory and antioxidant effect against various diseases, but its efficacy and activity are not yet analysed against the myocardial infarction. In this protocol, we tried to establish the correlation between the Boeravinone B on ISO‐induced MI injury in rats associated with the alteration in the gut microbiota. In the current investigation, we scrutinized the potential effect of Boeravinone B against ISO‐induced MI in rats and its underlying mechanism was also expounded based on the composition of gut microbiota, anti‐apoptotic, anti‐inflammatory and antioxidant activities.

## 2 MATERIAL AND METHODS

### 2.1 Chemical and reagents

Boeravinone B was purchased from Sigma‐Aldrich. All the chemicals and reagents used in the experimental study were analytical graded.

### 2.2 Experiment animal

Adult Sprague Dawley (230‐280 gm; sex – male) rats were used in the current experimental investigation. The experimental rats were collected from the animal house and housed in polypropylene cages (individual) under controlled temperature (22 ± 5°C) and humidity (60%‐70%) with alternate light and dark cycles. In the entire experimental period, the rats received the standard pellet and water ad libitum. This research was approved by Sichuan Provincial People's Hospital ethical committee (Approved No. 2016027).

### 2.3 Induction of myocardial infarction

Isoproterenol (ISO) was used in the induction of MI. To induce MI, ISO was dissolved in vehicle (saline) and 100 mg/kg dose subcutaneously injected into rats after every 24 hours for 2 days. The rats were sacrificed after 48 hours (after the 1st injection of ISO).^2^

### 2.4 Experimental protocol

The rats were divided into different groups with each group containing ten rats after the MI was successfully induced using ISO. Table 1 shows how the rats were divided into various classes.

**TABLE 1:** Showed the treatment

S. No	Group	Group name	Treatment
1	Group I	Normal	Saline 2 mL/Kg
2	Group II	ISO	100 mg/kg
3	Group III	ISO + BB	1.25 mg/kg
4	Group VI	ISO + BB	2.5 mg/kg
5	Group V	ISO + BB	5 mg/kg

All group rats were weighed, and blood samples were taken from the retro‐orbital plexus after the last BB treatment. To separate the serum, the blood samples were centrifuged at 15 000 rpm for 10 minutes. For further use, the separated serum was immediately stored at –20°C. At the end of the protocol, all group rats were euthanized using mild anaesthesia and heart tissue immediately removed, washed (saline), socked and finally weighed. A small piece (5 µm) of heart tissue was stored in the formaldehyde (40%) for histopathology. Rest of the heart tissue was kept at −20°C for biochemical estimation.

### 2.5 Nitric oxide and inducible nitric oxide

Nitric oxide (NO) and inducible nitric oxide synthase (iNOS) levels were estimated using the commercial kits following the manufacturing protocol (Nanjing Jiancheng Bioengineering Institute, Nanjing, China).

### 2.6 Lipid parameters

Lipid parameters, including triglyceride (TG), total cholesterol (TC), low‐density lipoprotein (LDL), very low‐density lipoprotein (VLDL) and high‐density lipoprotein (HDL) were estimated using the commercial kits following the manufacturing protocol (Nanjing Jiancheng Bioengineering Institute, Nanjing, China). The level of low‐density protein (LDL) and very low‐density lipoprotein (VLDL) was estimated using the given formula.

$$\text{LDL}\;\left(\text{mg/dL}\right)\;\text{= TC}‐\text{HDL}‐\left(\text{TG/5}\right)$$

VLDL=TG/5

### 2.7 Cardiac injury markers

The cardiac injury markers such as CK, TnT, CK‐MB and LDH were determined using commercial kits following the manufacturing protocol Nanjing Jiancheng Bioengineering Institute (Nanjing, China).

### 2.8 Estimation of cardiac function

Pressure volume catheter (1.9F; Scisense Instruments) was used for estimating the cardiac function using the previous method.^2^ To determine volume and pressure, the catheter was inserted into the carotid artery and then progressed into the left ventricle. Various cardiac function parameters such as left ventricular end‐diastolic pressure (LVEDP), stroke work (SW) and left ventricular end‐systolic pressure (LVESP). To calculate the region inside the pressure volume (PV) loop and estimate ventricular function, PV loops were then adjusted under the conditions of preload adjustment, which was elicited by occlusion of the inferior vena cava. The slope of end‐diastolic and end‐systolic volume points, which are load independent indices of myocardial ventricular and contractility compliance, was calculated using the end‐diastolic pressure volume relation (EDPVR) and end‐systolic pressure volume relation (ESPVR).^2^

### 2.9 Gene expression analysis

Ribonucleic acid (RNA) isolation kit (Promega) was used for isolating RNA from the heart following the manufacturing protocol. M‐MLV reverse transcriptase was used for performing the reverse transcription. SYBR Green qPCR SuperMix on a LightCycler^®^ 480 Real‐Time PCR system was used to quantify real‐time reverse transcription using 384‐well plates following the manufacturing instruction. All primer sequences are presented in Table 2. GAPDH was used as the internal standard.

**TABLE 2:** List of target gene primers

S. No	Gene	Sequence (5′‐3′)	
		Forwarded	Reverse
1	INFy	TGAGCATCGCCAAGTTCGAG	CCTTTTCCGCTTCCTTAGGCT
2	Tnfa	GTCCCAACAAGGAGGAGAAGT	TTTGCTACGACGTGGGCTAC
3	IL‐6	GCCCACCAGGAACGAAAGTC	TGGCTGGAAGTCTCTTGCGG
4	IL‐1β	CAGGATGAGGACCCAAGCAC	GTCGTCATCATCCCACGAGT
5	Cdx2	GGAAGCCAAGTGAAAACCAG	CTTTCCTCCTGATGGTGATG
6	MCP‐1	GCAGGTCTCTGTCACGCTTC	GGCATTAACTGCATCTGGCTGA
7	Tff3	ACCCTGCTGCTGGTCCTGGTTGCT	CCACAGTCCACCCTGACATTTGC
8	Bax	GGAGACACCTGAGCT GACCT	ATCCTCTGCAGCTCCATGTT
9	Bcl‐2	AGGATTGTGGCCTTCTTT GA	CAGATGCCGGTTCAGGTACT
10	Caspase‐3	GAGACAGACAGTGGAACTGACGATG	GGCGCAAAGTGACTGGATGA
11	Caspase‐9	AGCCAGATGCTGTCCCA TAC	ACCTGGGAAGGTGGAGTAGG
12	Chytochrome‐C	CCTTTGTGGTGTTGACCAGC	CCATGGAGGTTTGGTCCAGT
13	Klf3	GGCACAACGGTCAGATACGG	AGGAACCCAAGAGCCAAGAT
14	NGAL	ACATTCGTTCCAAGCTCCAG	TGGCAAACTGGTCGTAGTCA
15	Muc2	TCCCTCTTACAAGGGCAATG	TTCCAGCTGTTCCCAAAGTC
16	NFκB	GGGCTGACCTGAGTCTTCTG	GATAAGGAGTGCTGCCTTGC
17	Gapdh	TATAGCGAGAGGGACCCAGC	GACTCTCTTTGCACCACCCT

### 2.10 DNA extraction and 16s rRNA estimation

For isolating DNA from the stool, QIA‐amp FAST DNA Stool Mini Kit was used (Qiagen) following the manufacturing instruction. Briefly, faecal samples (200‐250 mg) were collected from the all‐group rats and homogenized in ASL lysis buffer (1000 μL) by vortexing for 2‐3 minutes in sterilized Eppendorf tube and incubated for 15 minutes in a water bath (85°C). After that, the homogenate was centrifuged for 5 minutes at 15 000 rpm and the supernatant was collected (residue was discarded). Collect the reddish‐yellow 600 μL supernatant and proteinase K (25 μL), AL solution (200 μL) were added and vortexed for next 15 seconds and incubated for 10 minutes in a water bath to maintain the temperature of 70°C. Finally, 1.5‐mL sterilized Eppendorf tubes were taken and the elution buffer (150 µL) was mixed with the columns and incubated at room temperature for the next 10 minutes and centrifuged for 1 minute at 14 000 rpm. Agarose gel electrophoresis and nanodrop methods were used for qualitative and quantitative analysis of the eluted DNA sample.

### 2.11 Haemodynamic estimation

BL‐420F Biological system (TME Technology Co., Ltd) was used for estimating haemodynamic variables using the previously reported method. Briefly, all group rats were anaesthetised and placed in the supine position. ECG devices were placed subcutaneously and connected to record the electrocardiograms. Pressure electricity transducer was used for estimating the ventricular contractile function with inserted Millar catheters into the left ventricular cavity through the right carotid artery. A catheter‐mounted pressure electricity transducer was used to obtain femoral arterial blood pressure readings.

### 2.12 Statistical analysis

GraphPad Prism 8 software (Graph Pad Software) was used for statistical analysis. For the statistical analysis, one‐way analysis of variance (ANOVA) was used for comparing the difference. Tukey multiple comparison test was performed for comparison of the different groups. The results were significantly considered if *P < .05, **P < .01 and ***P < .001.

## 3 RESULTS

### 3.1 Effect of BB drug on heart markers

**FIGURE 1:**
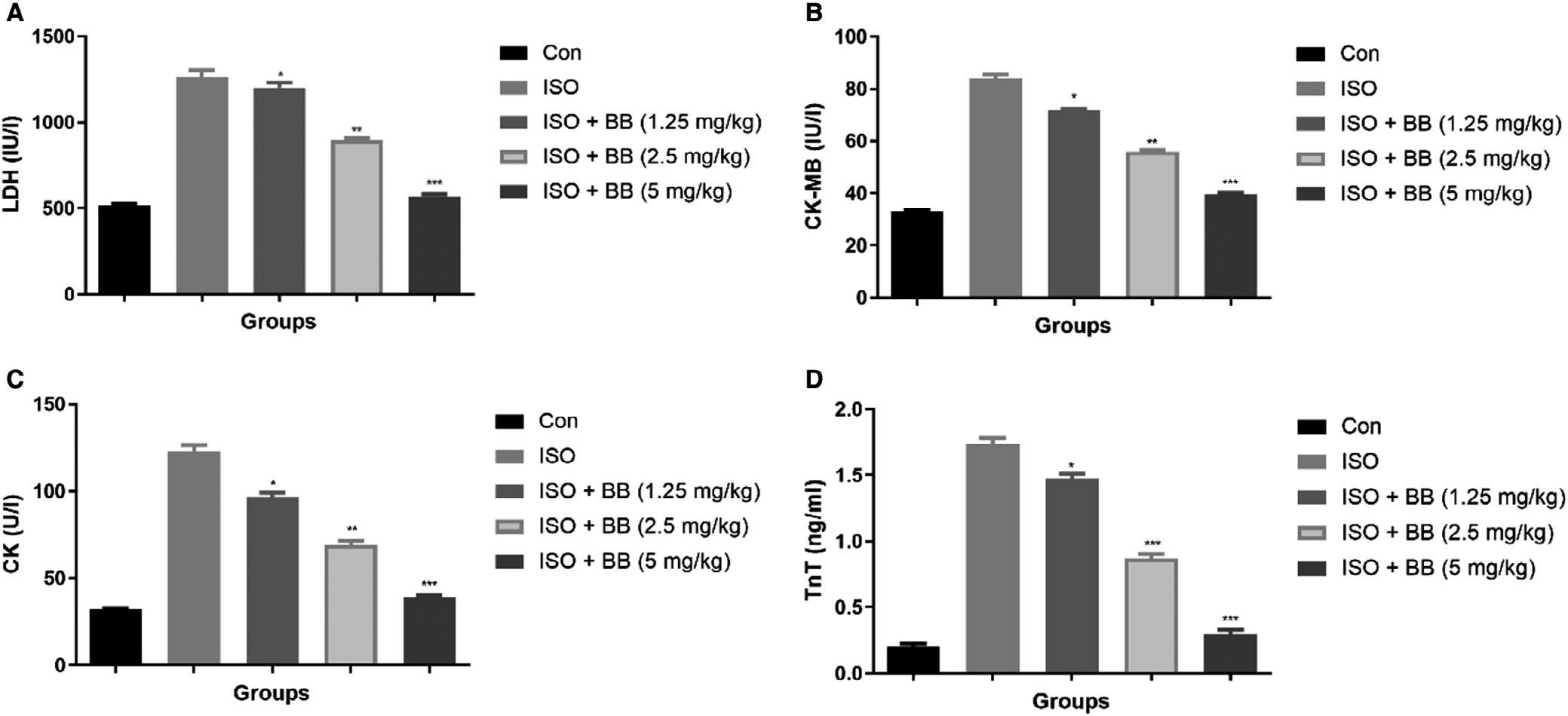
Effect of BB on heart marker level in the serum of tested and ISO‐induced MI rats. A, LDH, B, CK‐MB, C, CK and D, TnT. Tukey multiple comparison test was performed for the comparison the different groups. The results were significant considered if *P < .05, **P < .01 and ***P < .001

The level of heart markers was boosted as a result of the induction of MI. A similar momentum observed in this experimental protocol. When compared to control group rats, ISO‐induced MI rats showed the boosted level of LDH (Figure 1A), CK‐MB (Figure 1B), CK (Figure 1C) and TnT (Figure 1D). BB administration had effectively suppressed the heart injury induced by ISO by reducing the level of LDH, CK‐MB, CK and TnT in the serum (Figure 1). BB (5 mg/kg) treatment significantly (*P* < .001) down‐regulated the level of heart markers almost near to the level of heart markers in control group rats.

### 3.2 Effect BB on cardiac function parameters

**FIGURE 2:**
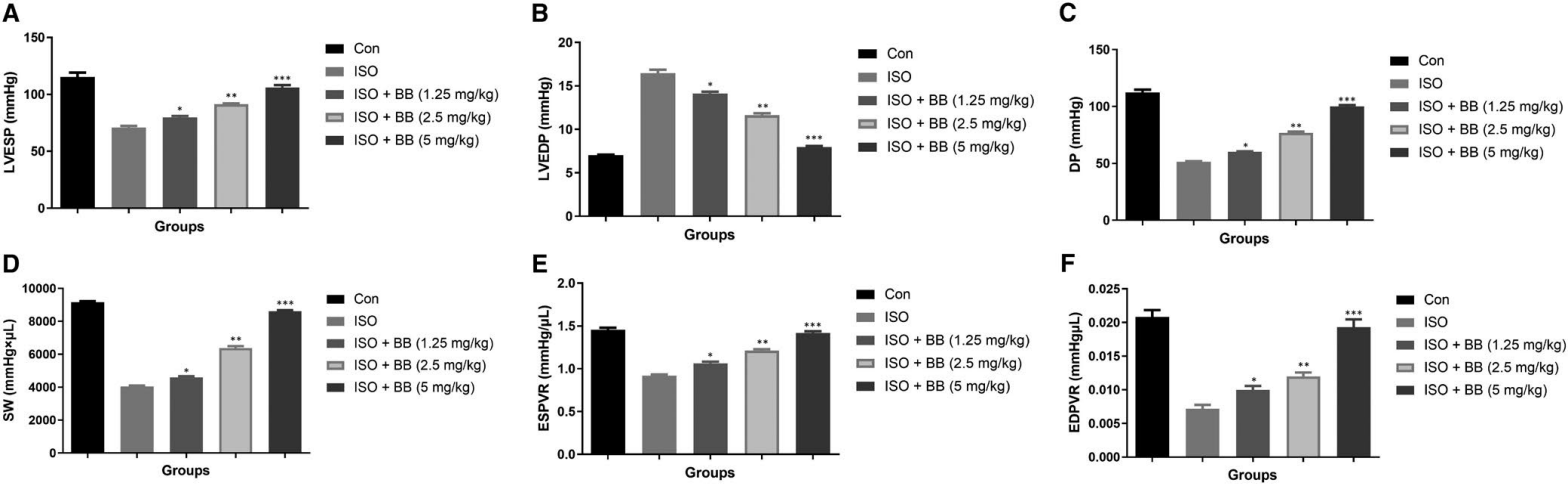
Effect of BB on cardiac function parameters in the tested and ISO‐induced MI rats. A, LVESP, B, LVEDP, C, DP, D, SW, E, EDPVR. Tukey multiple comparison test was performed for the comparison the different groups. The results were significant considered if *P < .05, **P < .01 and ***P < .001

Cardiac function parameters were estimated at the end of the experimental study. ISO‐induced MI rats showed the reduced level of LVSEP, DP, SW, ESPVR, EDPVR and increased level of LVEDP. BB treatment exhibited better cardiac protection by elevating the amounts of LVSEP, DP, SW, ESPVR, EDPVR and reducing the amount of LVEDP in ISO‐induced MI in rats. BB treatment considerably enhanced the cardiac diastolic and systolic function in the rats even after they were subjected to MI. (Figure 2).

### 3.3 Effect of BB on nitric oxide and inducible nitric oxide

**FIGURE 3:**
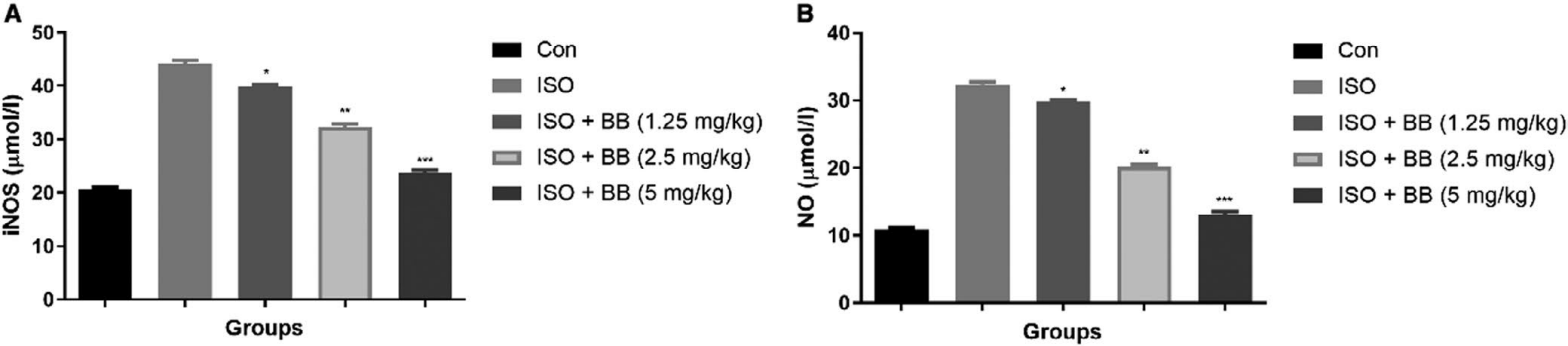
Effect of BB on the biochemical parameter of tested and ISO‐induced MI rats. A, iNOS and B, NO. Tukey multiple comparison test was performed for the comparison the different groups. The results were significant considered if *P < .05, **P < .01 and ***P < .001

Isoproterenol‐induced MI rats showed an increased level of NO and iNOS compared with normal control. BB suppressed the level of NO and iNOS at dose‐dependent manner (Figure 3).

### 3.4 Effect of BB on antioxidant enzymes

**FIGURE 4:**
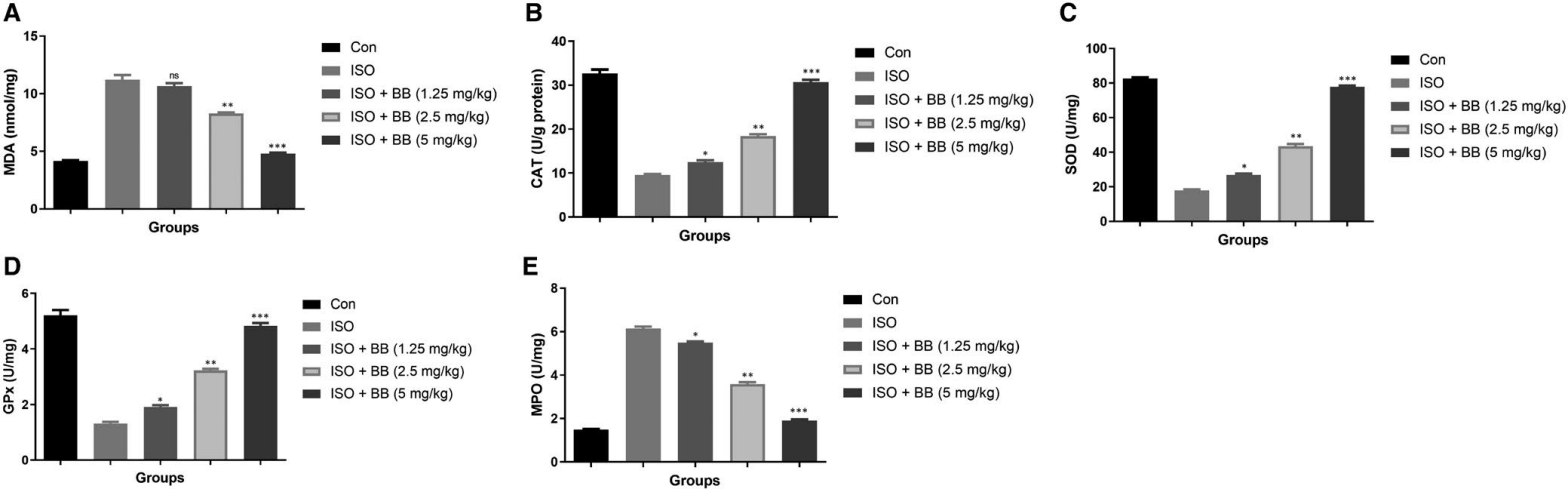
Effect of BB on antioxidant parameters in the tested and ISO‐induced MI rats. A, MDA, B, CAT, C, SOD, D, GPx and E, MPO. Tukey multiple comparison test was performed for the comparison the different groups. The results were significant considered if *P < .05, **P < .01 and ***P < .001

Antioxidant enzymes such as MDA (Figure 4A), CAT (Figure 4B), SOD (Figure 4C), GPx (Figure 4D) and MPO (Figure 4E) were estimated in the different groups of rats. ISO‐induced MI rats exhibited an increased level of MDA and reduced levels of CAT, SOD, GPx and MPO compared with control group rats. BB treatment considerably reduced the level of MDA and elevated the level of CAT, GPx, SOD and MPO.

### 3.5 Effect of BB on lipid parameter

**FIGURE 5:**
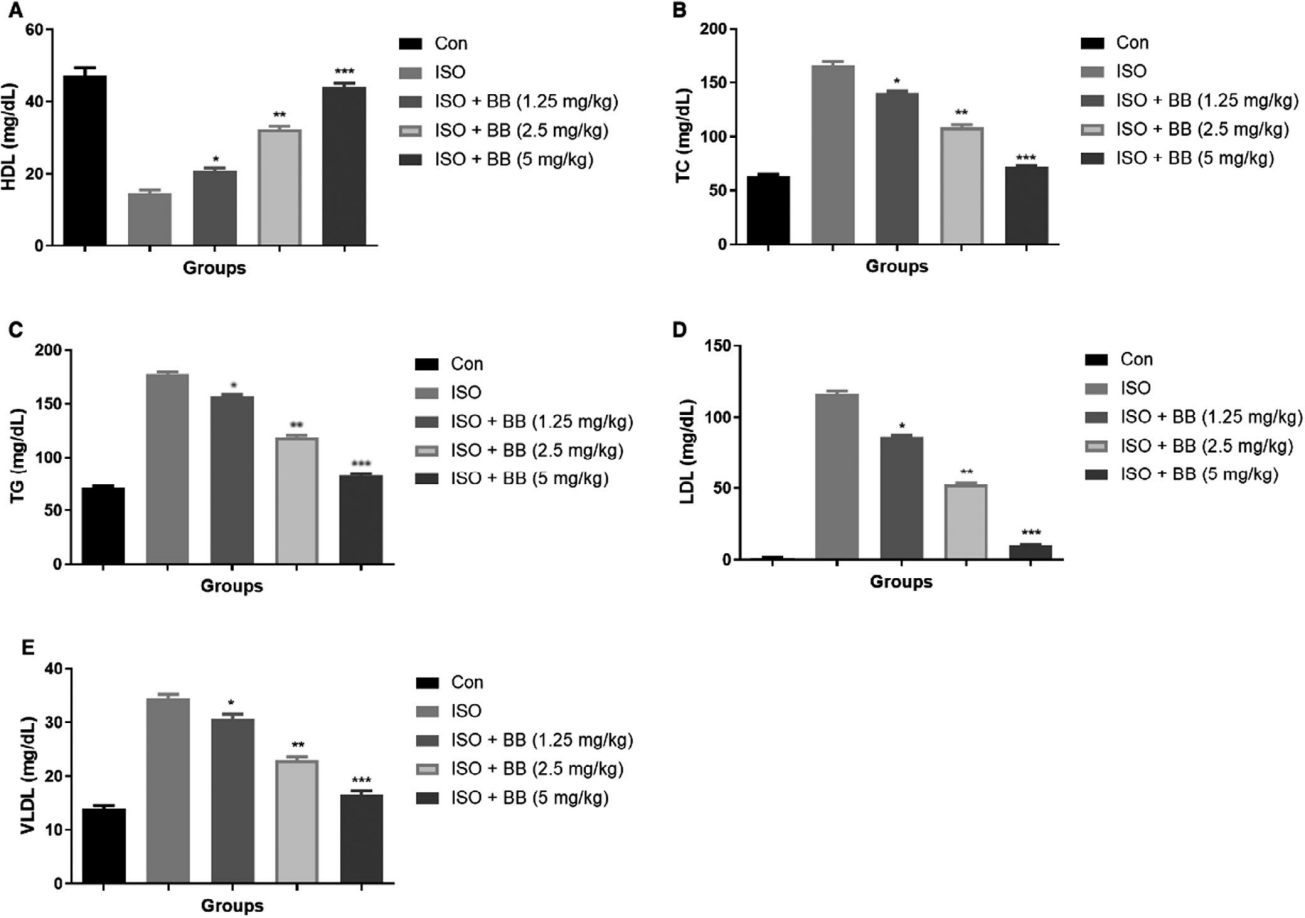
Effect of BB on lipid parameters in the tested and ISO‐induced MI rats. A, HDL, B, TC, C, TG, D, LDL and E, VLDL. Tukey multiple comparison test was performed for the comparison the different groups. The results were significant considered if *P < .05, **P < .01 and ***P < .001

The lipid profile such as TC, HDL, TG, LDL and VLDL estimated in all groups of rats. ISO‐induced MI rats showed the increased level of LDL, TC, VLDL, TG and reduced level of HDL. BB treatment significantly (*P* < .001) suppressed the level of LDL, TC, VLDL, TG and boosted the level of HDL (Figure 5).

### 3.6 Effect of BB on pro‐inflammatory cytokines

**FIGURE 6:**
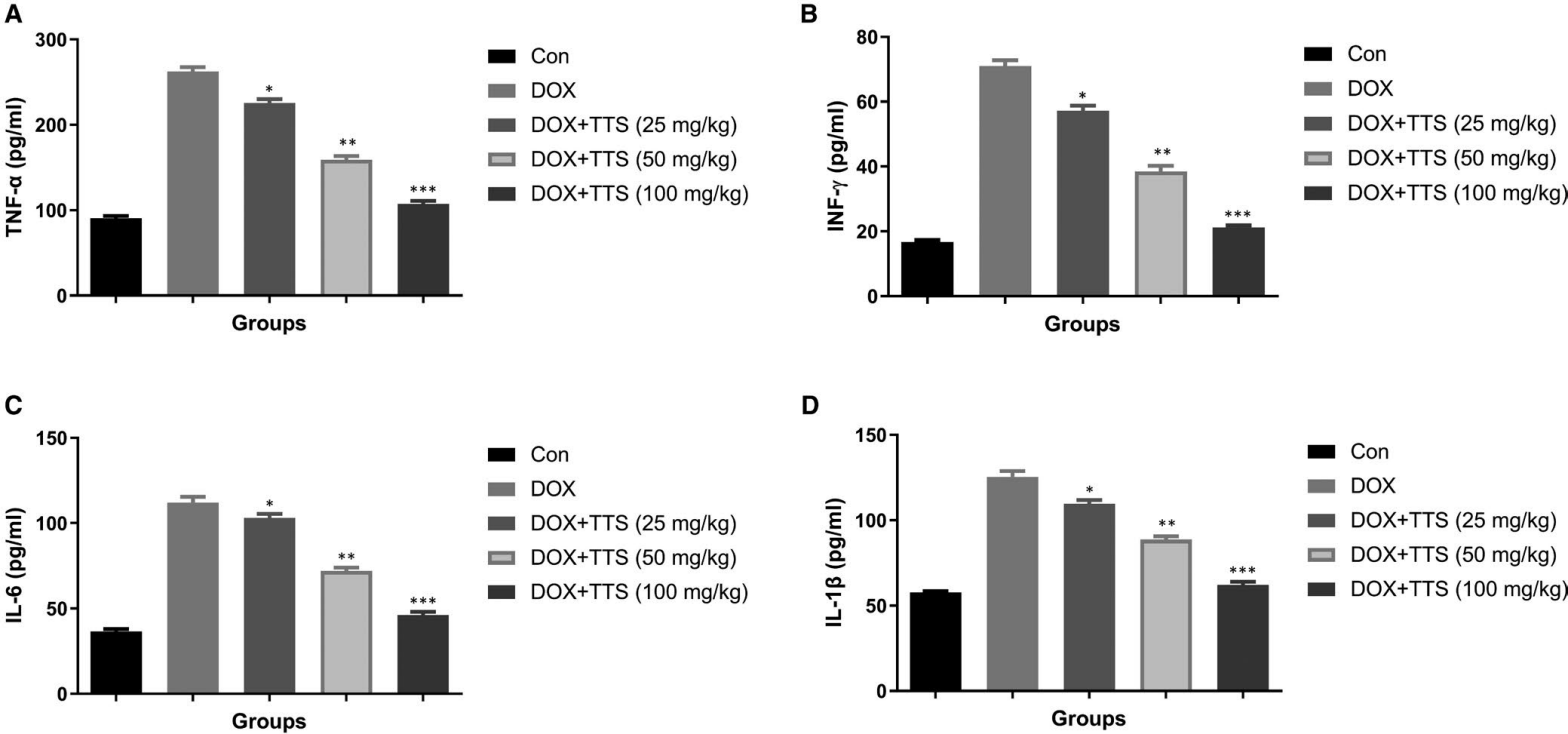
Effect of BB on pro‐inflammatory cytokines in the serum of tested and ISO‐induced MI rats. A, TNF‐α, B, INF‐γ, C, IL‐6 and D, IL‐1β. Tukey multiple comparison test was performed for the comparison the different groups. The results were significant considered if *P < .05, **P < .01 and ***P < .001

**FIGURE 7:**
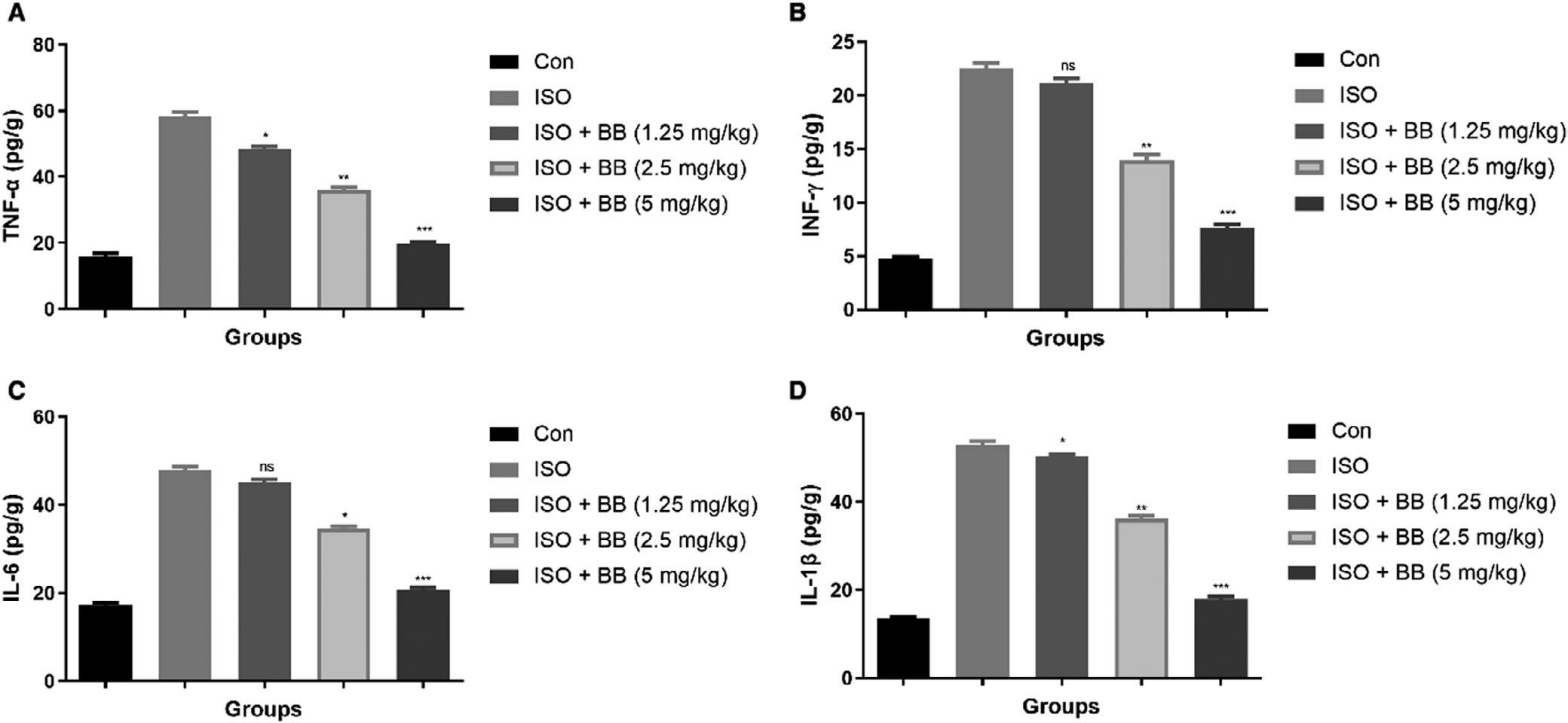
Effect of BB on pro‐inflammatory cytokines in the heart tissue of tested and ISO‐induced MI rats. A, TNF‐α, B, INF‐γ, C, IL‐6 and D, IL‐1β. Tukey multiple comparison test was performed for the comparison the different groups. The results were significant considered if *P < .05, **P < .01 and ***P < .001

Pro‐inflammatory cytokines include IL‐6, TNF‐α, INF‐γ and IL‐1β in the serum and heart tissue. ISO‐induced MI rats showed the boosted level of TNF‐α, INF‐γ, IL‐6 and IL‐1β in the serum (Figure 6) and heart tissue (Figure 7). BB treatment significantly (*P* < .001) suppressed the pro‐inflammatory cytokines in the serum as well as heart tissue.

### 3.7 Effect of BB on gene expression

**FIGURE 8:**
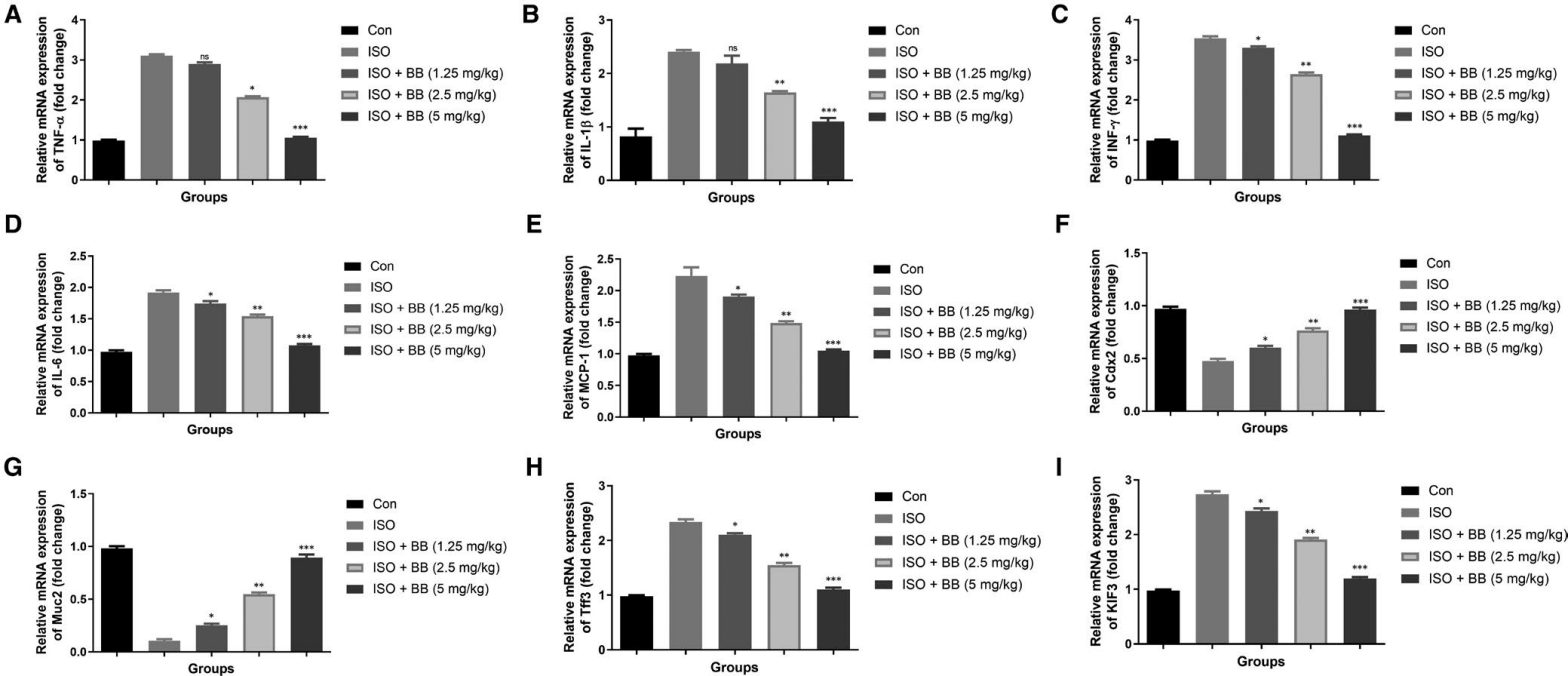
Effect of BB on mRNA expression of tested and ISO‐induced MI rats. A, TNF‐α, B, IL‐1β, C, INF‐γ, D, IL‐6, E, MCP‐1, F, Cdx2, G, Muc2, H, Tff3 and I, KIF3. Tukey multiple comparison test was performed for the comparison the different groups. The results were significant considered if *P < .05, **P < .01 and ***P < .001

In this experimental study, we analysed the pro‐inflammatory cytokine genes such as TNF‐α (Figure 8A), IL‐1β (Figure 8B), INF‐γ (Figure 8C), IL‐6 (Figure 8D) and MCP‐1 (Figure 8E) in the different groups of rats. The expression of pro‐inflammatory cytokine genes boosted in the ISO‐induced MI rats and BB treatment considerably reduced the pro‐inflammatory cytokines in a dose‐dependent manner. This finding suggests that BB having the ability to exert the cardioprotective effect in rats with ISO‐induced MI which may be attributed by the alteration of gut microbiota.

Isoproterenol‐induced MI rats showed the reduced expression of Cdx2 (Figure 8F), Muc2 (Figure 8G) and increased expression of Tff3 (Figure 8H) and KIF3 (Figure 8I) genes. ISO‐induced MI rats exhibited an increased level of Cdx2, Muc2 and suppressed level of Tff3, KIF3. BB treatment considerably down‐regulated the level of Cdx2 and Muc2 and up‐regulated the level of Tff3 and Kif3.

**FIGURE 9:**
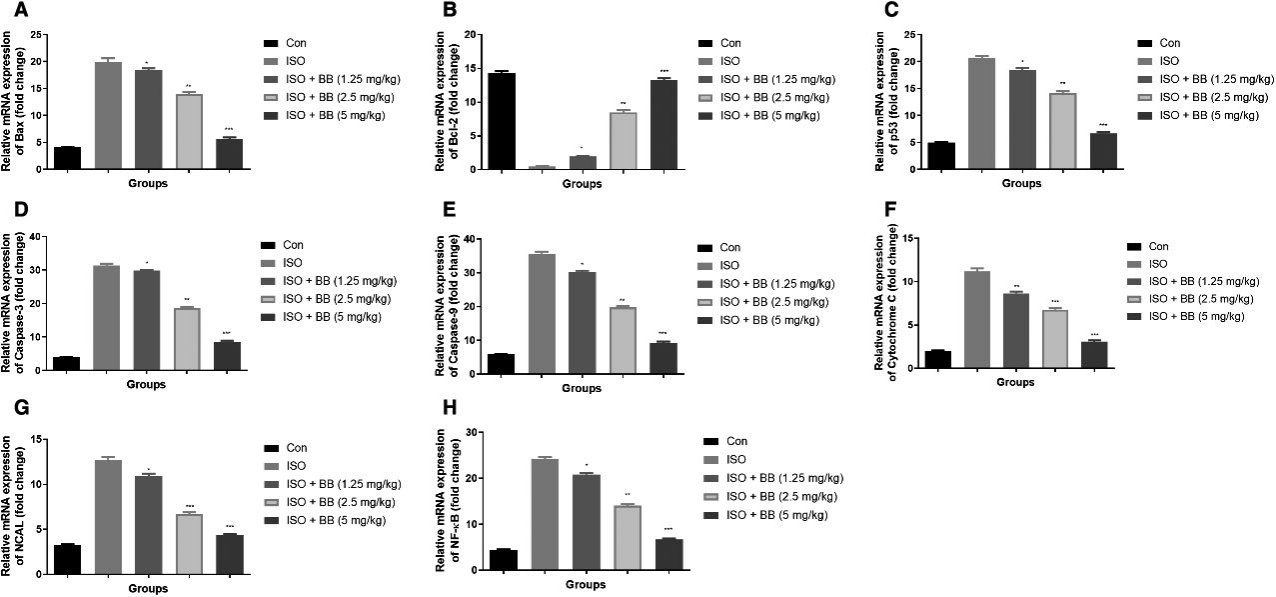
Effect of BB on mRNA expression of apoptosis and inflammatory parameter of tested and ISO‐induced MI rats. A, Bax, B, Bcl‐2, C, p53, D, caspase‐3, E, caspase‐9, F, cytochrome C, G, NCAL and H, NF‐κB. Tukey multiple comparison test was performed for the comparison the different groups. The results were significant considered if *P < .05, **P < .01 and ***P < .001

Figure 9 exhibited the effect of BB on the mRNA expression during inflammation and apoptosis. ISO‐induced MI rats showed an up‐regulation in the expression of p53, caspase‐3, Bax, caspase‐9, cytochrome C, NCAL and NF‐κB and down‐regulation in the expression of Bcl‐2. BB treatment reversed the expression by down‐regulating the level of p53, caspase‐3, Bax, caspase‐9, cytochrome C, NCAL and NF‐κB expressions and up‐regulating the level of Bcl‐2 expression level.

### 3.8 Effect of BB on gut microbiota

**FIGURE 10:**
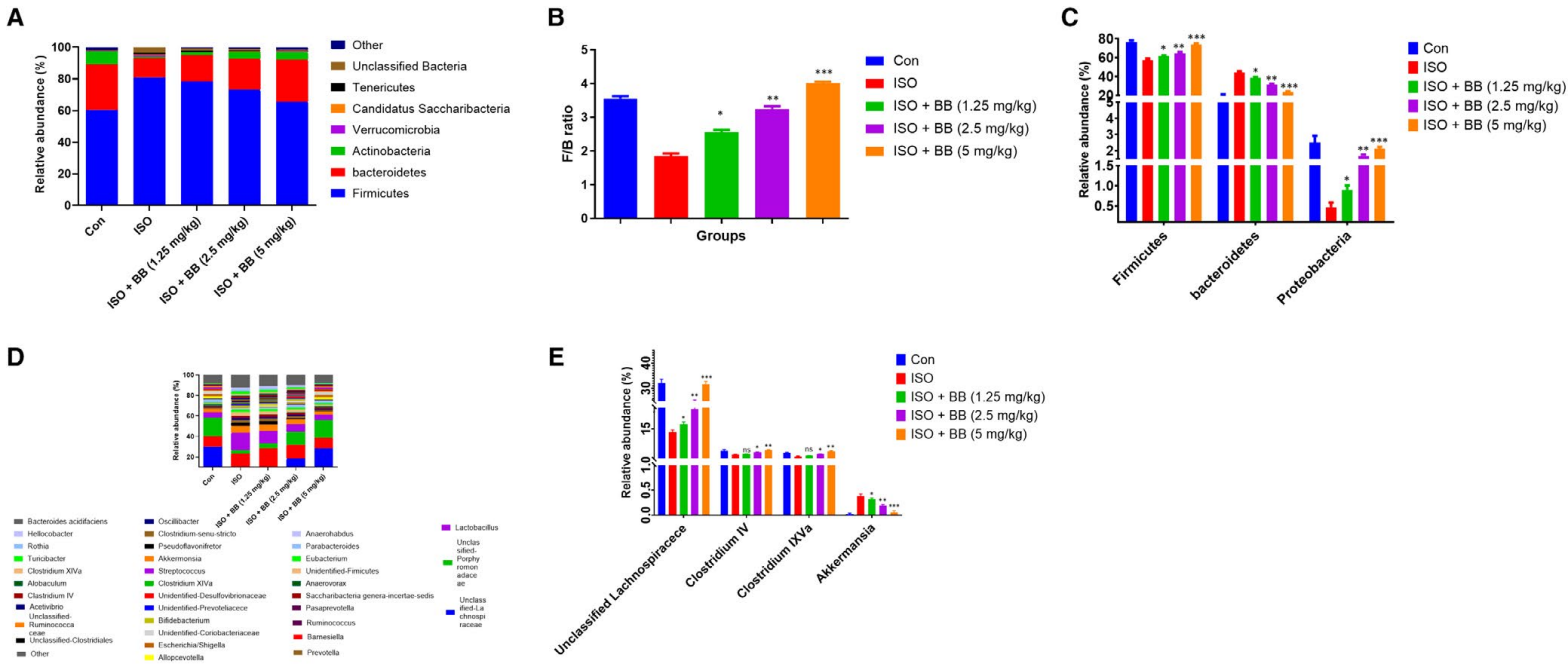
Effect of BB on gut microbiota following faecal microbiota transplantation of tested and ISO‐induced MI rats. A, composition of bacterial species, B, F/B ratio, C, Composition of bacterial species at the genus levels, D, relative abundance of mucin‐related genera and inflammation‐suppressing and E, Composition of bacterial species at the genus levels. Tukey multiple comparison test was performed for the comparison the different groups. The results were significant considered if *P < .05, **P < .01 and ***P < .001

The ability of BB to change the gut microbiome of faecal microbiota transplanted from the recipients was investigated at the end of the experimental study. In contrast to the standard control group rats, the ISO‐induced MI rats had a lower relative abundance of Firmicutes and a higher relative abundance of Bacteroidetes, as well as a lower F/B ratio (Figure 10A‐B). In ISO‐induced MI rats, the relative abundance of *Firmicutes, Proteobacteria* and *Bacteroidetes* was decreased, whereas the relative abundance of Bacteroidetes increased. The BB treatment increased the relative abundance of *Firmicutes, Proteobacteria* and *Bacteroidetes,* whereas it diminished the relative abundance of Bacteroidetes. The effect of BB on different genera of gut microbiota was shown in Figure 10D. Figure 10E demonstrated the reduction in the relative abundance of *Clostridium* IV, *CLoastridium* XIVa and Unclassified *Lachnospiraceae* and enhanced the *Akkermansia* relative abundance in ISO‐induced MI rats and BB‐treated rats reversed the relative abundance of gut microbiota.

### 3.9 Effect of BB on cardiac histopathology

**FIGURE 11:**
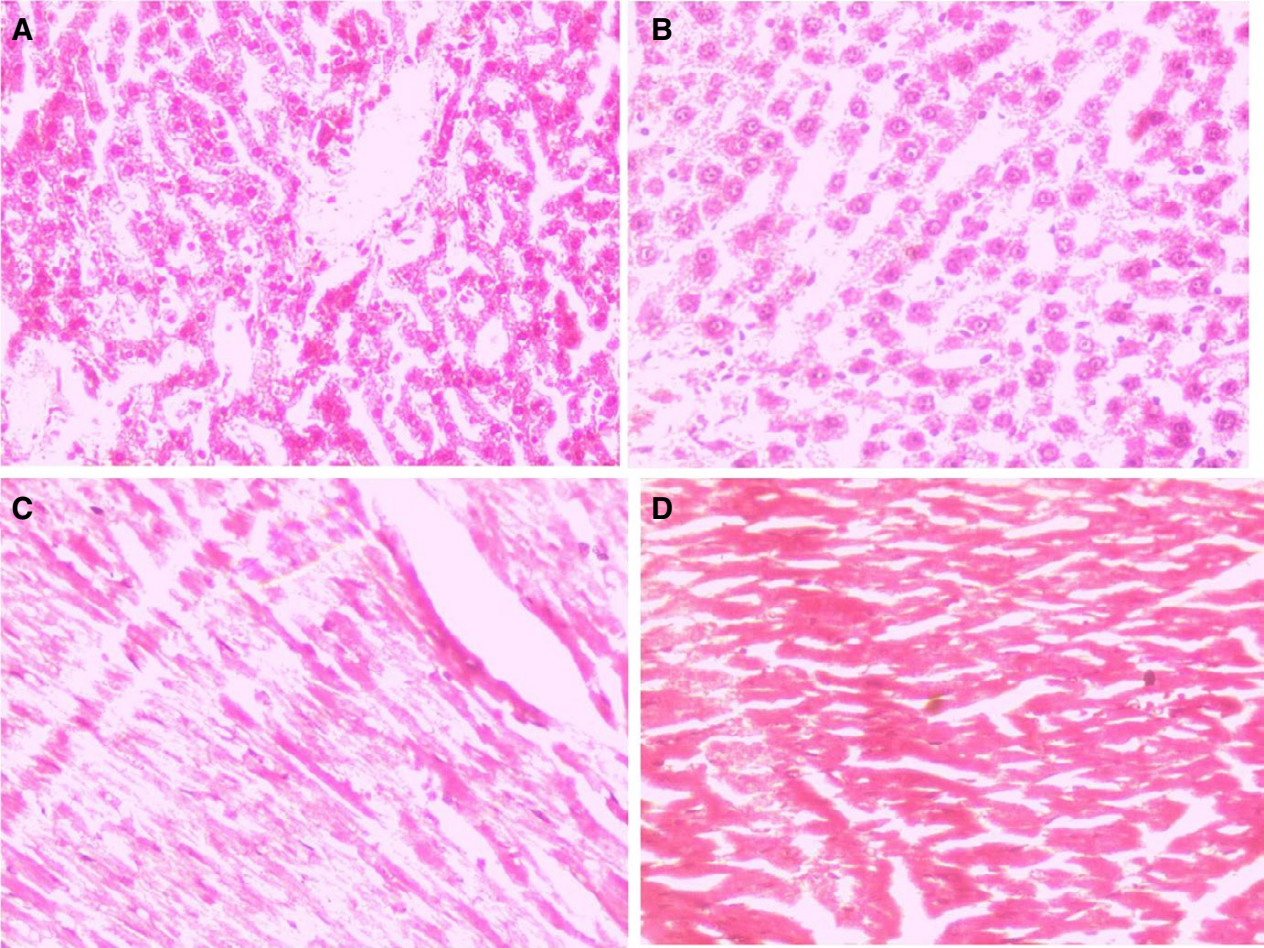
Effect of BB on the heart histopathology of tested and ISO‐induced MI rats. A, ISO‐induced MI rats, B, ISO + BB (2.5 mg/kg), C, ISO + BB (5 mg/kg) and D, ISO + BB (10 mg/kg)

Figure 11 shows the effect of BB on the cardiac histological alteration in the experimental rats. Normal control rats did not show any changes in the cardiac section and demonstrate the normal architecture with proper myofibrillar arrangement. ISO‐induced MI rats showed the expansion of necrosis and depicted disoriented myofibril in the cardiac histopathology with intense neutrophil infiltration. BB treatment considerably reduced the neutrophil infiltration with the less incidence of necrosis and illustrates the better myofibrillar arrangement.

## 4 DISCUSSION

Pre‐clinical studies suggest that the MI can be treated by specific plant‐based interventions. The plant‐based drugs have potent anti‐inflammatory and antioxidant activities against myocardial damage, and they are widely used as a result of its safety and efficacy. In this experimental study, we estimated the cardioprotective effect of Boeravinone B against ISO‐induced MI via the estimation of antioxidant and anti‐inflammatory effects and also the level and activity of gut microbiota was measured. ISO is a sympathomimetic amine like adrenaline that acts on adrenergic receptors. It increased cardiac performance relatively at a higher rate as a result the number of heart beats increased at a high rate when injected in rats. MI is classified primarily based on diastolic and systolic dysfunctions.^2^, ^3^

Exposure to ISO causes myocardial fibrosis in rats. Scar formation in myocardial tissue is often linked with the position and severity of ischaemia.^9^, ^10^ Previous research suggests that nitric oxide (NO) is a key molecule that significantly controls a variety of cardiac dysfunctions such as vasodilation, blood pressure, platelet and leucocyte adhesion and smooth muscle cell reduction. All the above‐mentioned cardiac dysfunctions cause scar formation in the myocardium.^1^, ^2^, ^10^ During an acute MI, the heart produces a large amount of NO, which worsens scar formation and also increases the heart weight and heart/bodyweight index. The recent study proved that the BB treatment decreased NO levels and suppressed heart/bodyweight index in rats. This result showed that BB may suppress the injury induced by MI in human beings too.

Isoproterenol has been related to an increase in serum lipids, which simultaneously leads to an increase in the risk of coronary heart disease.^4^, ^5^ ISO‐induced MI rats showed the boosted levels of LDL, TC, VLDL, TG and suppressed level of HDL, showing a positive relationship with myocardial infarction. ISO‐induced MI rats treated with BB showed a reduction in the serum lipid level and it slowly reached the normal level. Increased level of LDL and reduced level of HDL create a correlation with MI. The increased low density lipid (LDL) level could be attributed to an increase in lipid biosynthesis through cardiac cyclic adenosine monophosphate.^3^, ^14^ ISO group rats showed a boosted level of TG as a result of enhanced synthesis or could be as a result of the deposition of acyl CoA and amplified glycerol production via augmented glycolytic flux. ISO‐induced MI rats exhibited dysfunction in lipid parameters via different mechanisms, and BB treatment considerably improved the lipid profile and provided the cardioprotective effect.

Lactate dehydrogenase and CK‐MB serve as a significant biomarker of myocardial infarction (MI). During the MI, the level of LDH and CK‐MB boosted as a result of the expansion of diseased condition.^9^, ^10^ The current study showed the elevated levels of LDH and CK‐MB in the ISO‐induced MI group rats, and the levels of LDH and CK‐MB were suppressed by BB treatment which considerably decreased myocardial necrosis. Other cardiac biomarkers such as TnT is a contractile protein commonly present in cardiac muscle and commonly used for estimating myocardial dysfunction, especially in MI patients.^4^ ISO‐induced rats showed an up‐regulation in the level of TnT and thereby BB treatment considerably down‐regulated the level of TnT and suggesting a better protection against cardiac injury. BB treatment considerably enhanced the suggested myocardial ability through improved LVSP and myocardial contractility as estimated in terms of ‐dp/dtmax and + dp/dtmax.^21^, ^22^ The above‐mentioned results suggest the cardioprotective effect of BB against the ISO‐induced MI in rats by preventing necrosis and thereby it enhances the myocardial function.

Isoproterenol treatment considerably boosted the level of NO. Studies suggest that the NO and iNOS activities increased during the MI. The activation of NO and iNOS is boosted as a result of enhanced adrenergic receptors.^23^, ^24^ The increased amount of NO, combined with the multiple oxygen reactive species and causing nitrosative stress, results in the development of massive reactive oxidant peroxynitrite (ONOO‐) species, which can interfere with tissue repair mechanisms. Suppression of superoxide expression can reduce peroxynitrite activity, which may be beneficial in the treatment of MI.^25^, ^26^ The activity of iNOS was found to be higher in the ISO‐induced MI group rats and BB treatment considerably suppressed the activity of iNOS and suggested the protective effect against MI.

Caspase‐3 is the most important member of the protease family, and it is called as the executioner of apoptosis. Caspase‐3 is a useful marker for determining whether apoptosis‐related genes are activated in myocardial injury. According to previous research, activation of the caspase‐3 gene enhances apoptosis during penetrating myocardial injury.^22^, ^24^ ISO‐induced MI group rats exhibited increased expression of caspase‐3 protein, and its expression was considerably suppressed by BB treatment. Previous research indicates that cell death plays an important role in MI pathogenesis because the apoptotic pathway and ISO‐induced MI are interconnected. During the induction of MI by ISO, there was an increased expression of pro‐apoptotic signalling proteins such as cytochrome C, P53, caspase‐3, Bax, caspase‐9 and reduced expression of anti‐apoptotic proteins such as Bcl‐2.^22^, ^23^, ^24^ During the apoptotic event, oxidative stress alters the lysosomes and mitochondria. Lysosomes and mitochondria are involved in the process of apoptosis through the activation of pro‐apoptotic and pro‐caspases. During apoptotic stimuli, apoptotic promoters such as Bax (Bcl‐2 family) induce oxidative stress, activate cytochrome C secretion and enhance cell death. Anti‐apoptotic members, such as Bcl‐2 (a member of the Bcl‐2 family), play a critical role in the control of apoptotic pathways by scavenging free radicals within cells and reducing cytochrome C secretion into the cytoplasm.^22^, ^25^, ^26^ Both caspase enzymes, caspase‐3 and caspase‐9, are involved in the pathophysiology of apoptosis. The cytochrome C secreted into the cytoplasm by the activation of proteolytic cleavage of pro‐caspase‐9 and activated caspase‐3, pro‐caspase‐9 and caspase‐9. It is well known that caspase‐3 activation significantly induces the final stage of apoptosis. Previous research showed the relationship between MI and apoptosis.^2^, ^4^, ^5^ In this experiment, we found that the BB treatment decreased Bax and caspase‐3 expression and simultaneously increased Bcl‐2 expression in ISO‐induced MI rats. The results showed that the BB treatment can decrease the apoptosis induced by ISO in rat heart effectively by suppressing the expression of caspase‐3, Bax, p53 and caspase‐9 and enhances the Bcl‐2 expression.

Lipocalin‐2 is the member of lipocalin family protein. During normal conditions, the level of NGAL is observed to be lower in the heart, lungs, kidneys, stomach, etc But, during ischaemia, inflammation, acute kidney injury and intoxication, the level of NGAL is boosted. Few reports suggest that NGAL is not only considered as a biomarker for renal injury, but also considered as a potentially valuable marker for MI.^1^, ^2^, ^5^ The level of NGAL is boosted in the chronic heart failure patients compared with the normal individuals. Moreover, the investigation suggests that the increased level of NGAL is related to the inflammatory reaction.^5^, ^11^ In our experimental research, it has been proved that the increased expression of NGAL improved the expression of pro‐inflammatory cytokines such as IL‐6, IL‐1β, IL‐8 and TNF‐α.

Additionally, the evidence suggests that ISO increases the secretion of numerous chemokines and inflammatory cytokines that induce the NF‐κB. The NF‐κB plays a significant role in the regulation of inflammatory cytokines and boosting the targeted genes (IL‐6 and IL‐1β) transcription, resulting in an increase in the inflammatory response induced by the activation of pro‐inflammatory cytokines.^1^, ^7^, ^14^ IL‐1β can boost the chemotaxis of neutrophils and inflammatory mediators, which induce tissue injury and inflammatory reactions. The IL‐1β mRNA expression is related to the degree of inflammation and can also be used as an indicator of disease and severity. The reduction of IL‐1β is notable for anti‐inflammatory properties leading to the suppression of the cascade and activating the pro‐inflammatory cytokines, iNOS and NF‐κB. Other pro‐inflammatory cytokines including IL‐6 involved in the various inflammatory processes include neutrophils maturation and recruitment.^1^, ^7^, ^14^ During the vascular endothelial cell toxicity, the level of IL‐6 is boosted, and it starts the catalase activity and augments the toxic and inflammatory effects and ultimately induces the cell damage. Our result clearly indicated that the BB treatment considerably suppressed the expression of pro‐inflammatory cytokines and inflammatory mediators.

The removal of gut microbiota effectively eliminated ISO‐induced injury in various organs, including the heart, which clearly states that gut microbiota plays a key role in the expansion and progression of MI in the heart caused by ISO.^16^, ^27^ Various dietary supplements already prove their protective effect against ISO‐induced cardiac toxicity by targeting the gut microbiota. In this study, we estimated the cardioprotective effect of BB on the ISO‐induced MI and also, we found that the BB treatment precludes the deleterious effect of gut dysbiosis including alteration of gut microbiota, gut leakiness and inflammation. Faecal microbiota transplantation involved in the gut microbiome and involved in improving cardiomyopathy and also involved in modulating the dysregulation induced by ISO in rats.

As a result, altering homeostasis by targeting the gut microbiota may be a novel way to treat ISO‐induced MI. In ISO‐induced MI rats, the findings showed that the BB administration prevents gut microbiota dysbiosis and the deterioration of intestinal barrier integrity.^16^, ^27^, ^28^ After cardiotoxicity, the structure of the gut microbiota decreases in richness and diversity and administration of dietary supplements modified the gut microbiota and helped in suppressing the cardiotoxicity.^28^, ^29^ In this experiment, we observed that the BB treatment significantly decreased and prevented changes in gut microbiota alpha richness and diversity. In ISO‐induced MI rats, BB treatment increased microbiota richness and prevented alpha diversity reduction. Furthermore, in ISO‐induced MI rats, BB treatment preserved the composition of the gut microbiota and protected the relative abundance of unique bacterial genera and phyla. BB treatment significantly increased the number of Bacteroidetes and Firmicutes, as well as the F‐to‐B ratio, a commonly used biomarker of gut microbiota dysbiosis, implying that BB treatment effectively rebalanced or preserved the ISO‐induced gut microbiota dysbiosis to a usual or safe state.

The cardiac failure pathogenesis is the hot topic of research in the scientific community. In this experimental study, we observed that Firmicutes (composed 84 species), belong to the Clostridium genera.^16^, ^27^ Previous research suggests that modulation in Firmicutes is related to the induction of cardiovascular disease lifetime.^16^, ^29^ ISO‐induced MI rats exhibited an increased level of Firmicutes, suggesting that the GUT microbiota interacts with various signalling pathways such as the SCFAs and trimethylamine (TMA)/trimethylamine N‐oxide (TMAO) pathway. Bacteroidetes is another significant phylum, playing a critical role in normal functioning of the heart.^17^, ^30^ BB treatment considerably reduced the relative abundance of Firmicutes and suggested the cardioprotective effect. The different species of Firmicutes, such as *Coprococcus 2, Lachnospiraceae, Ruminococcus and Tyzzerella* are associated with various cardiovascular complications.

Furthermore, BB treatment increased the levels of *Lachnospiraceae, Clostridium* clusters IV, *Roseburia* and *Roseburia*, all of which produce anti‐inflammatory metabolites that may help to suppress the changes in pH level in the gut and inhibit excessive bacterial growth, as well as they serve as biotherapeutics to treat inflammatory diseases.^17^, ^28^, ^30^ *Oscillibacter* and *Butyicicoccus* level were considerably boosted after the BB treatment. Both the bacteria exhibited anti‐inflammatory potential and played an important role in intestinal defence barrier by boosting the mucins production and TJ protein expression, leading to higher villi and longer nutrient absorption capacity.^29^, ^30^ BB treatment significantly increased the relative abundance of unclassified Clostridiales, which produces butyrate and has been shown to inhibit NLRP1 inflammasome activation and prevent inflammatory bowel disease (IBD). As a result, ISO‐induced MI rats had a higher degree of *Akkermansia*, and BB treatment significantly decreased *Akkermansia* abundance. The nutrient source Akkermansia (intestinal mucin‐degrading bacterium) is widely used to host secreted mucus O‐glycans (MOGs), which causes the mucus barrier to erode. The mucus layer is in a state of flux, oscillating between regeneration via goblet cells and deprivation via gut bacteria.^27^, ^29^ If bacterial absorption of mucin‐derived nutrients exceeds new intake, the integrity of this vital barrier can be jeopardized, triggering an inflammatory response and increasing gut permeability. The abundance of various positively anti‐inflammatory bacteria was significantly increased after BB therapy, indicating that it had a beneficial effect on MI in rats.

In this research, we discovered that in ISO‐induced MI rats, reduced expression of Cdx2 and Muc2 and an increase in the number of goblet cells in the villi, which release glycoproteins, implying a weakness in mucus production capability as the secretory role of goblet cells, are exhausted.^2^, ^22^ This exhaustion may be as a result of continuous secretion of mucin‐2 glycoprotein in large amounts from the goblet cells. BB treatment continuously maintained the goblet cell number and Akkermansia relative abundance, which further boosted the protection and repairment of mucus barrier.

## 5 CONCLUSION

The current study found that administering BB to rats reduced dysregulation and gut dysbiosis during ISO‐induced myocardial infarction. BB treatment significantly reduced the heart parameters at dose‐dependent manner. ISO treatment significantly reduced endogenous antioxidant enzymes and boosted the inflammatory reaction. BB treatment considerably altered the antioxidant enzymes and inflammatory mediators.

- Boeravinone B treatment reduced the level of MDA and increased the level of SOD, CAT and GPx.
- Boeravinone B treatment reduced the inflammatory reaction in the serum and heart tissue by suppressing of pro‐inflammatory cytokines and inflammatory mediators in the serum and heart tissues.
- Boeravinone B treatment altered the cardiac function parameters.
- Boeravinone B treatment alleviated gut microbiota by maintaining the F/B ratio.
- Boeravinone B treatment also maintained the relative abundances of *Clostridium IV, Butyricicoccus, Clostridium XIVs, Akkermansia* and *Roseburia*.
- Boeravinone B treatment considerably regulates gut microbiota and suggests the cardioprotective effect.

## CONFLICT OF INTEREST

All authors declare no conflict of interest.

## AUTHOR CONTRIBUTION

**Yu Chen:** Conceptualization (equal); Formal analysis (equal); Investigation (equal); Writing‐original draft (equal); Writing‐review & editing (equal). **Lei Peng:** Data curation (equal); Formal analysis (equal); Software (equal); Validation (equal); Writing‐review & editing (equal). **Shaoqing Shi:** Data curation (equal); Formal analysis (equal); Funding acquisition (equal); Investigation (equal); Project administration (equal). **Gang Guo:** Project administration (equal); Resources (equal); Software (equal); Supervision (equal); Validation (equal); Visualization (equal); Writing‐original draft (equal); Writing‐review & editing (equal). **Heling Wen:** Methodology (lead); Supervision (equal); Writing‐original draft (equal); Writing‐review & editing (equal).

## Data Availability

The data that support the findings of this study are available from the corresponding author upon reasonable request.
