# Corrigendum

**DOI:** 10.1111/jcmm.17357

**Published:** 2022-06-06

In Chen et al,^1^ the published article contains errors in Figure 6 and Figure 10. The correct figures are shown below.

**FIGURE 6:**
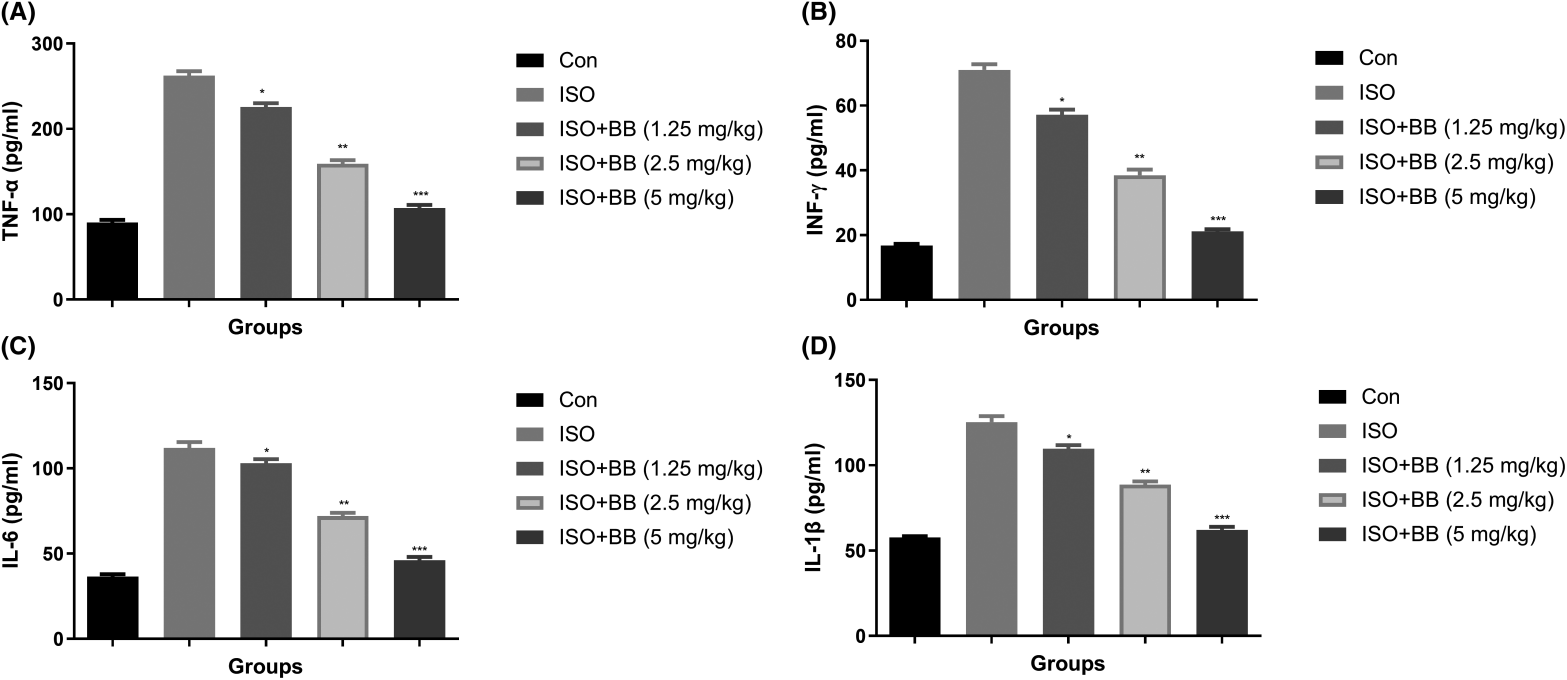
Effect of BB on pro‐inflammatory cytokines in the serum of tested and ISO‐induced MI rats. (A) TNF‐α, (B) INF‐γ, (C) IL‐6 and (D) IL‐1β. Tukey multiple comparison test was performed for the comparison the different groups. The results were significantly considered if *p < 0.05, **p < 0.01 and ***p < 0.001

**FIGURE 10:**
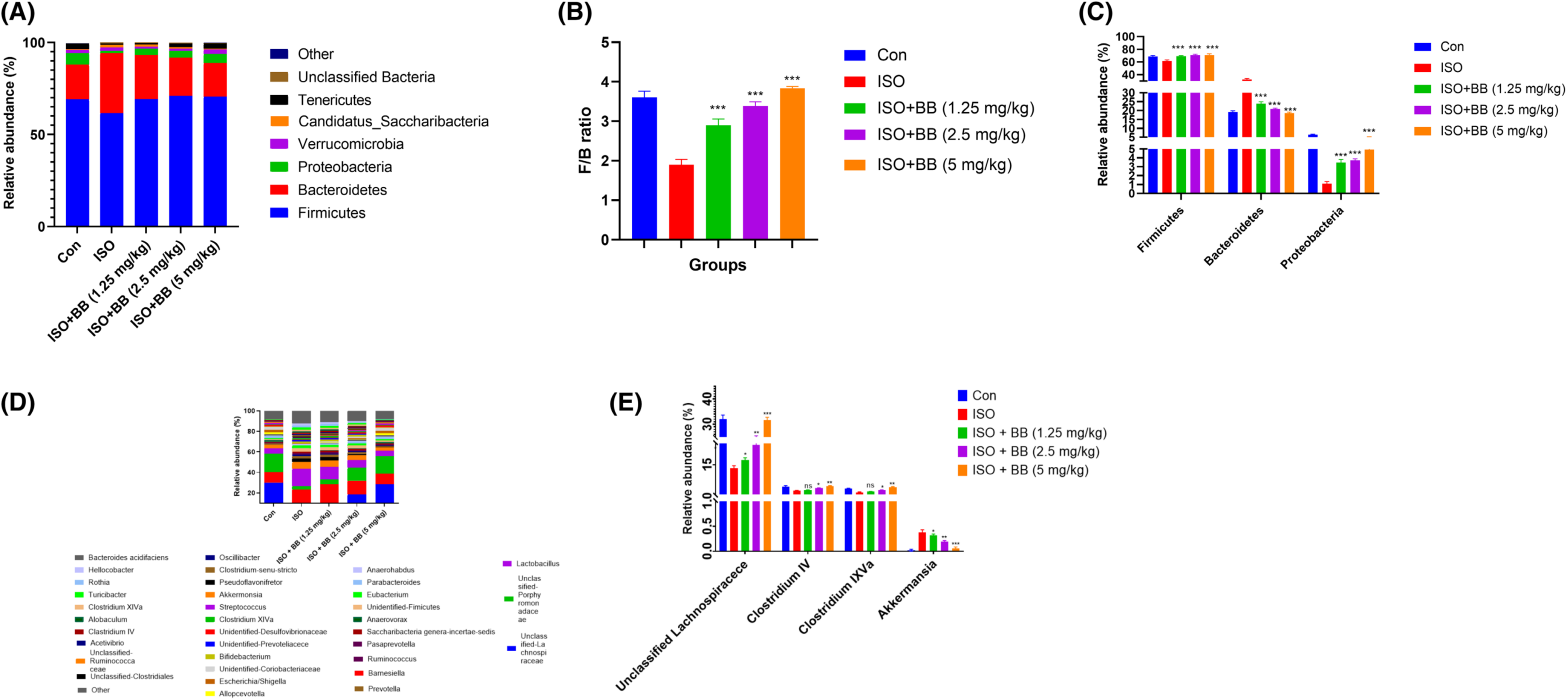
Effect of BB on gut microbiota of tested and ISO‐induced MI rats. (A) composition of bacterial species, (B) F/B ratio, (C) composition of bacterial species at the genus levels, (D) relative abundance of mucin‐related genera and inflammation‐suppressing and (E) composition of bacterial species at the genus levels. Tukey multiple comparison test was performed for the comparison the different groups. The results were significantly considered if *p < 0.05, **p < 0.01 and ***p < 0.001

The authors regret inadvertent errors in the description of the analysis in the original manuscript as follows. In the ‘3.7 Effect of BB on gene expression’ of results section, we wrote: ‘ISO‐induced MI rats exhibited an increased level of Cdx2, Muc2 and suppressed level of Tff3, KIF3. BB treatment considerably down‐regulated the level of Cdx2 and Muc2 and up‐regulated the level of Tff3 and Kif3’. This description was incorrect and should have read instead: ‘ISO‐induced MI rats exhibited an increased level of Cdx2, Muc2 and suppressed level of Tff3, KIF3 after BB treatment’. In the ‘3.8 Effect of BB on gut microbiota’ of results section, we wrote: ‘The ability of BB to change the gut microbiome of faecal microbiota transplanted from the recipients was investigated at the end of the experimental study. In contrast to the standard control group rats, the ISO‐induced MI rats had a lower relative abundance of *Firmicutes* and a higher relative abundance of *Bacteroidetes*, as well as a lower F/B ratio (Figure 10A,B). In ISO‐induced MI rats, the relative abundance of *Firmicutes, Proteobacteria* and *Bacteroidetes* was decreased, whereas the relative abundance of *Bacteroidetes* increased. The BB treatment increased the relative abundance of *Firmicutes, Proteobacteria* and *Bacteroidetes*, whereas it diminished the relative abundance of *Bacteroidetes’*. This description was incorrect and should have read instead: ‘The ability of BB to change the gut microbiome was investigated at the end of the experimental study. In contrast to the standard control group rats, the ISO‐induced MI rats had a lower relative abundance of *Firmicutes* and a higher relative abundance of *Bacteroidetes*, as well as a lower F/B ratio (Figure 10A,B). In ISO‐induced MI rats, the relative abundance of *Firmicutes* and *Proteobacteria* was decreased, whereas the relative abundance of *Bacteroidetes* increased. The BB treatment increased the relative abundance of *Firmicutes* and *Proteobacteria*, whereas it diminished the relative abundance of *Bacteroidetes’*. In the legend of Figure 11, we wrote: ‘Effect of BB on the heart histopathology of tested and ISO induced MI rats. (A) ISO induced MI rats, (B) ISO + BB (2.5 mg/kg), (C) ISO + BB (5 mg/kg) and (D) ISO + BB (10 mg/kg)’. It should have read instead: ‘Effect of BB on the heart histopathology of tested and ISO induced MI rats. (A) ISO induced MI rats, (B) ISO + BB (1.25 mg/kg), (C) ISO + BB (2.5 mg/kg) and (D) ISO + BB (5 mg/kg)’. The authors confirm all key experimental findings and conclusions of this article remain unchanged.

The authors apologize for the inconvenience this may cause.
